# A Meta-Analysis of Risk Factors for Transient and Permanent Hypocalcemia After Total Thyroidectomy

**DOI:** 10.3389/fonc.2020.614089

**Published:** 2021-02-24

**Authors:** Yuan Qin, Wei Sun, Zhihong Wang, Wenwu Dong, Liang He, Ting Zhang, Hao Zhang

**Affiliations:** Department of Thyroid Surgery, The First Hospital of China Medical University, Shenyang, China

**Keywords:** transient hypocalcemia, permanent hypocalcemia, risk factors, total thyroidectomy, meta-analysis

## Abstract

**Background:**

As hypocalcemia is the most common complication of total thyroidectomy, identifying its risk factors should guide prevention and management. The purpose of this study was to determine the risk factors for postthyroidectomy hypocalcemia.

**Methods:**

We searched PubMed, Web of Science and EMBASE through January 31, 2019, and assessed study quality using the Newcastle–Ottawa Scale.

**Results:**

Fifty studies with 22,940 patients met the inclusion criteria, of which 24.92% (5716/22,940) had transient hypocalcemia and 1.96% (232/11,808) had permanent hypocalcemia. Significant (*P* < 0.05) predictors of transient hypocalcemia were: younger age, female, parathyroid autotransplantation (PA), inadvertent parathyroid excision (IPE), Graves’ disease (GD), thyroid cancer, central lymph node dissection, preoperative severe Vitamin D deficiency, preoperative Vitamin D deficiency and a lower postoperative 24 h parathyroid hormone (PTH) level. Preoperative magnesium, preoperative PTH and Hashimoto’s thyroiditis were not significant predictors of transient hypocalcemia. IPE, GD, and thyroid cancer were associated with an increased rate of permanent hypocalcemia, but gender and PA did not predict permanent hypocalcemia.

**Conclusion:**

Important risk factors for transient and permanent hypocalcemia were identified. However, given the limited sample size and heterogeneity of this meta-analysis, further studies are required to confirm our preliminary findings.

## Highlights

A meta-analysis of 50 studies and 22,940 patients identified significant clinical and biochemical predictors of postthyroidectomy transient and permanent hypocalcemia.

## Introduction

Postoperative hypocalcemia is the most common complication and often the most troubling long-term consequence of total thyroidectomy (TT) (1). Thyroid surgeons must employ strategies to prevent postthyroidectomy hypocalcemia and minimize its effects. By definition, transient hypocalcemia occurs less than 6 months after surgery, while permanent hypocalcemia persists for 6 months or longer after surgery (2). The incidence rates of transient and permanent hypocalcemia ranged from 3.15% to 64.25% and from 0% to 6.84%, respectively, in the studies included in this meta-analysis **(** **Table 1**). In many cases, hypocalcemia is mild and manifests with symptoms of peripheral paresthesia, muscle cramps and anxiety. However, the condition is sometimes severe and can lead to acute life-threatening conditions, such as tetany, laryngospasm, confusion, seizures, arrhythmias and heart failure (3). Although most cases are transient and self-limiting, up to 10% can become permanent, and result in the need for lifelong calcium and vitamin D supplementation, repeated clinic visits and an increased risk of long-term complications (4).

**Table 1:** Characteristics of the included studies (N = 50).

Author	Year	Country	Malignant Rate	Study design	Transient Hypocalcemia		Permanent Hypocalcemia		QualityAssessment of study	Author	Year	Country	Malignant Rate	Study design	Transient Hypocalcemia		Permanent Hypocalcemia		QualityAssessment of study
					Proportion	Rate	Proportion	Rate							Proportion	Rate	Proportion	Rate	
Arman S	2018	UK	39.3%	Retrospective	42/196	21.43%	7/196	3.57%	8	Griffin TP	2014	Ireland	33.9%	Retrospective	29/121	23.97%			7
Brophy C	2018	Ireland	25.4%	Retrospective	69/173	39.88%			7	Lorente-Poch L	2014	Spain	22.4%	Prospective	278/657	42.31%			7
Eismontas V	2018	Lithuania	21%	Prospective	257/400	64.25%			7	Noureldine SI	2014	USA	53%	Retrospective	159/304	52.30%	2/304	0.66%	6
Falch C	2018	Germany	7.8%	Retrospective	160/702	22.79%	48/702	6.84%	7	Julián MT	2013	Spain	22.9%	Prospective	44/70	62.86%			7
Luo H	2018	China	NA	Retrospective	262/559	46.87%			7	Lang BH	2013	China	9.3%	Prospective	39/281	13.88%			7
Mazotas IG	2018	USA	49.4%	Retrospective	102/591	17.26%			6	Nair CG	2013	India	2.1%	Retrospective	190/806	23.57%	13/806	1.61%	7
Vasileiadis I	2018	UK	32.2%	Retrospective	549/2556	21.48%			7	Paek SH	2013	Korea	100%	Retrospective	135/531	25.42%	19/531	3.58%	6
Docimo G	2017	Italy	17.1%	Prospective	48/328	14.63%			7	Pisanu A	2013	Italy	45.5%	Prospective	33/112	29.46%	2/112	1.79%	7
Luo H	2017	China	98%	Retrospective	82/304	26.97%			7	Hallgrimsson P	2012	Sweden	0	Prospective	71/209	33.97%			8
Sahli Z	2017	USA	38.1%	Prospective	107/218	49.08%			7	Lang BH	2012	China	11.1%	Prospective	17/117	14.53%			7
Sitges-Serra A	2017	Spain	100%	Prospective	86/170	50.59%			6	Lin Y	2012	USA	100%	Retrospective	70/152	46.05%			7
Wang X	2017	China	100%	Retrospective	124/237	52.32%			7	Chapman DB	2011	USA	61.5%	Retrospective	22/52	42.31%			8
Wang X2	2017	China	100%	Retrospective	99/186	53.23%			7	Kirkby-Bott J	2011	UK	NA	Retrospective	44/165	26.67%			6
Applewhite MK	2016	USA	49.6%	Retrospective	300/554	54.15%			8	Welch KC	2011	USA	0	Retrospective	250/394	63.45%			7
Cherian AJ	2016	India	NA	Retrospective	67/150	44.67%			6	Sitges-Serra A	2010	Spain	22.4	Retrospective	222/442	50.23%	17/442	3.85%	8
Lang BH	2016	China	0	Prospective	130/569	22.85%	15/569	2.64%	7	Asari R	2008	Austria	42.4	Prospective	43/170	25.29%	2/170	1.18%	7
Mahmoud RR	2016	Brazil	0	Retrospective	54/142	38.03%			7	Erbil Y	2007	Turkey	0	Prospective	32/130	24.62%			7
Manatakis DK	2016	Greece	30.6%	Retrospective	48/281	17.08%			7	Manouras A	2007	Greece	16.2%	Retrospective	16/508	3.15%	0/508	0	7
Kala F	2015	Turkey	10%	Prospective	31/100	31.00%			7	Chiang FY	2006	China	0	Retrospective	37/107	34.58%			7
Kim WW	2015	Korea	100%	Retrospective	171/267	64.04%			6	Lombardi CP	2006	Italy	31.3%	Prospective	199/523	38.05%			7
Lee GH	2015	Korea	100%	Prospective	52/134	38.81%			7	Roh JL	2006	Korea	82.6%	Prospective	34/92	36.96%	3/92	3.26%	7
Miah MS	2015	UK	0	Prospective	48/225	21.33%			6	Serpell JW	2006	Australia	17.9%	Retrospective	131/334	39.22%	6/334	1.80%	7
Puzziello A	2015	Italy	4%	Retrospective	28/75	37.33%			7	Palazzo FF	2005	Australia	19.9%	Retrospective	149/1196	12.46%	10/1196	0.84%	8
Al-Khatib T	2014	Saudi Arabia	38%	Retrospective	42/213	19.72%			7	Lombardi CP	2004	Italy	42%	Retrospective	16/53	30.19%			7
Edafe O	2014	UK	26.1%	Retrospective	69/238	28.99%			7	Thomusch O	2003	Germany	3.6%	Prospective	429/5846	7.34%	88/5846	1.51%	7
										**Total**					**5716/22940**	**24.92%**	**232/11808**	**1.96%**	

Several predictors of hypocalcemia have been proposed in recent decades. Among these, age, gender, parathyroid injuries (e.g., contusion, blood supply disorder and inadvertent parathyroid excision), Vitamin D deficiency (VDD) and parathyroid hormone (PTH) levels have been suggested as the most important predictors of transient hypocalcemia (5). Medical and surgical strategies to minimize postthyroidectomy hypocalcemia include optimizing vitamin D levels, preserving parathyroid blood supply and autotransplanting ischemic parathyroid glands. However, studies have reported contradictory results. Therefore, accurate judgement and identification of risk factors for postoperative hypocalcemia are necessary to reduce the incidence of hypocalcemia.

Our meta-analysis of 50 studies aimed to analyze potential risk factors for postoperative transient and permanent hypocalcemia, and to prevent the development of clinical symptoms related to hypocalcemia by determining the reasons for these risks.

## Materials and Methods

Our systematic review was based on the preferred reporting items for systematic reviews and meta-analyses (PRISMA) statement (6) (**Supplementary Material Table 1**).

### Search Strategy

A comprehensive search of the relevant literature was performed using the PubMed, Web of Science and EMBASE databases for studies published through January 31, 2019 with the key words {[(hypocalc*) OR low calcium] AND thyroidectomy}. Two of the study’s authors (YQ and WS) conducted the selection process independently, and all disagreements were resolved by discussion.

### Selection Criteria

We included studies that met the following criteria: a) prospective or retrospective original research; b) English language; c) all of the patients underwent TT with or without lymph node dissection; and d) complete medical records were available for data extraction. The following exclusion criteria were used to eliminate studies: a) reviews, letters, abstracts or conference proceedings, articles with the full text unavailable in English, animal studies or irretrievable articles and b) patients who had a prior/concomitant parathyroidectomy, known hyperparathyroidism or preoperative hypocalcemia.

### Data Extraction and Quality Assessment

The two authors of the study (mentioned earlier) abstracted relevant data from the included articles in accordance with a prepared standardized form. Author(s), year of publication, country of study, study design, case number, surgical intervention, 13 possible risk factors and the number of the patients were recorded independently. The risk factors included age, gender, parathyroid autotransplantation (PA), inadvertent parathyroid excision (IPE), Graves’ disease (GD), Hashimoto’s thyroiditis (HT), thyroid cancer, central lymph node dissection (CLND), preoperative severe Vitamin D deficiency (SVDD), preoperative VDD, preoperative magnesium, preoperative PTH, and postoperative 24 h PTH.

There is no internationally accepted definition of hypocalcemia; hence, studies were included irrespective of the definitions used (**Supplementary Material Table 2**). Transient hypocalcemia occurs less than 6 months after surgery, while permanent hypocalcemia persists for 6 months or longer after surgery (2). According to the American Endocrine Society’s clinical practice guideline definition, serum 25OHD levels < 10 ng/ml (25 mmol/L) was defined as SVDD, and levels < 20 ng/ml (50 mmol/L) was defined as VDD (7).

Two investigators independently evaluated the quality of the included studies independently using the Newcastle–Ottawa Scale (NOS). The NOS is used to assess individual studies’ risk for bias in three domains: i) study group selection, ii) comparability of groups, and iii) assessment of outcomes or exposure. The total NOS score ranges from 0 to 9, and those with scores ≥ 6 are considered high quality studies with a low risk for bias (8).

### Statistical Analyses

All of the statistical analyses were performed using Review Manager (RevMan) software, Version 5.3 (The Nordic Cochrane Centre, Copenhagen). The results are presented as odds ratios (ORs) with 95% confidence intervals (CIs), and a *P*-value < 0.05 was considered statistically significant, except where otherwise specified. Heterogeneity was quantified using the Q-test and the I^2^ statistic. When *P* > 0.1 and I^2^ < 50%, a fixed-effects model was used; otherwise, a random-effects model was applied. Possible publication bias was tested using Begg’s funnel plot (**Supplementary Materials Figures S1** and **S2**).

## Results

**Figure 1:**
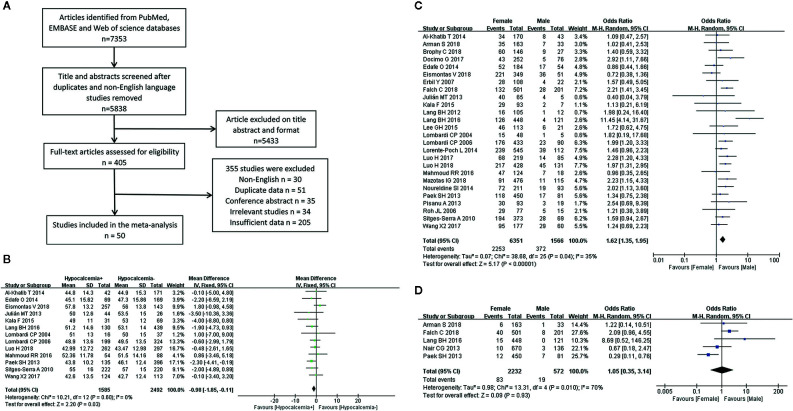
Flowchart of the meta-analysis **(A)** and the meta-analysis results for the incidence of hypocalcemia shown between the two groups; **(B)** Age for transient hypocalcemia; **(C)** Gender for transient hypocalcemia; **(D)** Gender for permanent hypocalcemia.

The systematic search of the literature yielded 7,353 studies, which were initially included in the meta-analysis; 1,515 studies were excluded because of duplication or the use of languages other than English. The titles and abstracts were read carefully, and another 5,433 articles that did not satisfy the requirements of an original research study; (reviews, case reports, letters and irrelevant studies were excluded). The full texts of the remaining 405 articles were evaluated and 50 (**Supplementary Material Table 2**) studies that met the inclusion criteria were selected for this meta-analysis (**Figure 1A**). Of the 22,940 included patients, 5,716 had postthyroidectomy hypocalcemia. The results of our meta-analysis showed a wide range of differences in the incidence rates of transient and permanent hypocalcemia among the included studies (range, 3.15%–64.25% and 0%–6.84%, respectively) (**Table 1**), with a total incidence of 24.92% (5,716/22,940) and 1.96% (232/11,808). The basic characteristics of the included studies are summarized in **Table 1**. The pooled outcomes of all items in this meta-analysis are presented in **Table 2**.

**Table 2:** Pooled outcomes of all the subgroups.

Risk factors	No. of studies	Statistical model	I^2^	P value	MD/OR	95% CI	P value
**Transient Hypocalcemia**							
Age	13	fixed-effects	0%	0.6	MD -0.98	(-1.85– -0.11)	**0.03**
Gender	26	fixed-effects	35%	0.04	OR 1.62	(1.35–1.95)	**<0.00001**
PA	17	fixed-effects	32%	0.1	OR 1.93	(1.68–2.22)	**<0.00001**
IPE	17	fixed-effects	13%	0.3	OR 2.46	(2.17–2.78)	**<0.00001**
GD	17	fixed-effects	0%	0.53	OR 1.89	(1.6–2.23)	**<0.00001**
Cancer	17	fixed-effects	31%	0.11	OR 1.46	(1.25–1.72)	**<0.00001**
CLND	16	fixed-effects	0%	0.79	OR 1.78	(1.49–2.11)	**<0.00001**
HT	5	fixed-effects	0%	0.76	OR 0.95	(0.72–1.26)	0.74
Preoperative SVDD	8	fixed-effects	38%	0.13	OR 1.64	(1.26–2.15)	**0.0002**
Preoperative VDD	8	random-effects	67%	0.004	OR 1.83	(1.12–2.99)	**0.02**
Preoperative magnesium	5	random-effects	86%	<0.0001	MD -0.02	(-0.06–0.01)	0.23
Preoperative PTH	9	fixed-effects	0%	0.62	MD -0.86	(-2.58–0.85)	0.32
Postoperative 24 h PTH	5	random-effects	60%	0.04	MD -19.82	(-22.48– -17.16)	**<0.00001**
**Permanent Hypocalcemia**							
Gender	5	random-effects	70%	0.01	OR 1.05	(0.35–3.14)	0.93
PA	4	fixed-effects	0%	0.5	OR 1.25	(0.72–2.18)	0.42
IPE	4	fixed-effects	0%	0.53	OR 4.35	(3.00–6.30)	**<0.00001**
GD	6	fixed-effects	29%	0.22	OR 2.25	(1.32–3.82)	**0.003**
Cancer	4	fixed-effects	0%	0.79	OR 2.07	(1.10–3.88)	**0.02**

MD, mean difference; OR, odds ratio; CI, confidence interval; PA, parathyroid autotransplantation; IPE, inadvertent parathyroid excision; GD, Graves’ disease; CLND, central lymph node dissection; HT, Hashimoto’s thyroiditis; SVDD, severe vitamin D deficiency; VDD, vitamin D deficiency; PTH, parathyroid hormone. The differences were deemed statistically significant at p < 0.05.

### Age

A fixed-effects model was used for this analysis (I^2^ = 0%, *P* = 0.60). Age was identified as a risk factor for transient hypocalcemia in 13 of the included studies, and younger age was found to be associated with an increased rate of hypocalcemia in patients who underwent TT (**Figure 1B**).

### Gender

The analysis of transient hypocalcemia included 26 studies and used a fixed-effects model (I^2^ = 35%, *P* = 0.04), while the analysis of permanent hypocalcemia included 5 studies and used a random-effects model (I^2^ = 70%, *P* = 0.01). Female patients who underwent TT had a higher incidence of transient hypocalcemia (**Figure 1C**), but the incidence of permanent hypocalcemia between the males and females was not significant (**Figure 1D**).

### Parathyroid Autotransplantation

**Figure 2:**
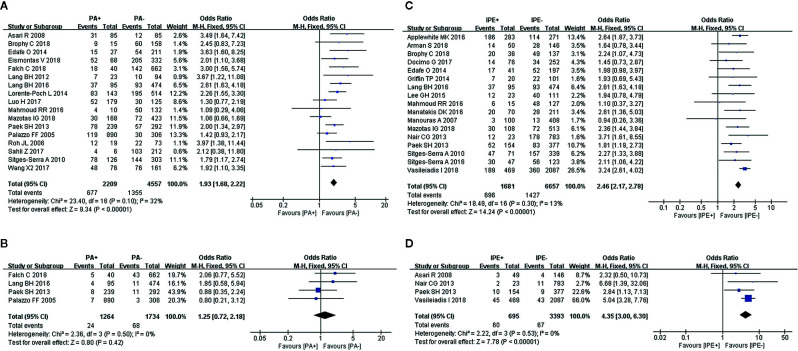
Meta-analysis results for the incidence of hypocalcemia between the two groups; **(A)** PA for transient hypocalcemia; **(B)** PA for permanent hypocalcemia; **(C)** IPE for transient hypocalcemia; and **(D)** IPE for permanent hypocalcemia.

Fixed-effects models were used to analyze the risks for transient (I^2^ = 32%, *P* = 0.1) and permanent hypocalcemia (I^2^ = 0%, *P* = 0.5) in patients who had undergone PA. The results showed that patients who underwent PA exhibited a 1.93-fold increased risk for transient hypocalcemia (**Figure 2A**), but PA was not associated with permanent hypocalcemia (**Figure 2B**).

### Inadvertent Parathyroid Excision

IPE was analyzed in 17 and 4 studies, respectively, as a risk factor for transient (I^2^ = 13%, *P* = 0.3) and permanent hypocalcemia (I^2^ = 0%, *P* = 0.53), using fixed-effects models. IPE significantly increased the incidence of transient (**Figure 2C**) and permanent hypocalcemia (**Figure 2D**).

### Graves’ Disease

**Figure 3:**
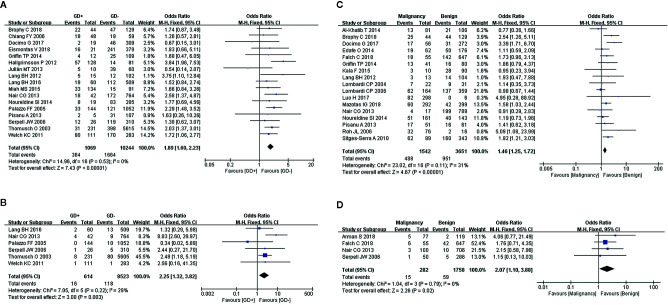
Meta-analysis results for the incidence of hypocalcemia between the two groups; **(A)** GD for transient hypocalcemia; **(B)** GD for permanent hypocalcemia; **(C)** thyroid cancer for transient hypocalcemia; and **(D)** thyroid cancer for permanent hypocalcemia.

We used a fixed-effects model for the analyses of transient (I^2^ = 0%, *P* = 0.53) and permanent hypocalcemia (I^2^ = 29%, *P* = 0.22), in 17 and 6 studies, respectively. The results showed that patients with GD who underwent TT exhibited a significantly higher incidence of transient (**Figure 3A**) and permanent hypocalcemia (**Figure 3B**).

### Cancer

A diagnosis of cancer in patients with transient and permanent hypocalcemia was assessed in 17 and 4 studies, respectively. Fixed-effects models were used for the analyses of both transient (I^2^ = 31%, *P* = 0.11) and permanent hypocalcemia (I^2^ = 0%, *P* = 0.79). We found that thyroid cancer was associated with increased rates of transient (**Figure 3C**) and permanent hypocalcemia in patients who underwent TT (**Figure 3D**).

### Central Lymph Node Dissection

**Figure 4:**
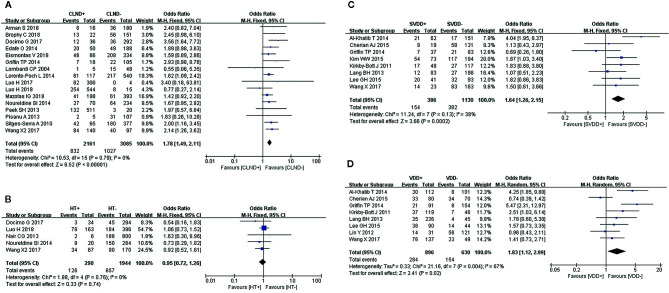
Meta-analysis results for the incidence of transient hypocalcemia between the two groups; **(A)** CLND for transient hypocalcemia; **(B)** HT for transient hypocalcemia; **(C)** preoperative SVDD for transient hypocalcemia; and **(D)** preoperative VDD for transient hypocalcemia.

Sixteen studies were included in the analysis of CLND using a fixed-effects model (I^2^ = 0%, *P* = 0.79),. We found a significant association between patients who underwent CLND and an increase in transient hypocalcemia (**Figure 4A**).

### Hashimoto’s Thyroiditis

We used a fixed-effects model to analyze HT (I^2^ = 0%, *P* = 0.76), which was identified as a risk factor for transient hypocalcemia in five studies. However, HT was not associated with transient hypocalcemia (**Figure 4B**).

### Preoperative Severe Vitamin D Deficiency

A fixed-effects model was used to analyze this risk factor (I^2^ = 38%, *P* = 0.13). Eight studies reported a significantly higher incidence of transient hypocalcemia in patients with preoperative SVDD (**Figure 4C**).

### Preoperative Vitamin D Deficiency

We used a random-effects model to analyze preoperative VDD (I^2^ = 67%, *P* = 0.004), which was identified as a risk factor for transient hypocalcemia in eight studies. The incidence of transient hypocalcemia was significantly increased in patients with VDD (**Figure 4D**).

### Preoperative Magnesium

**Figure 5:**
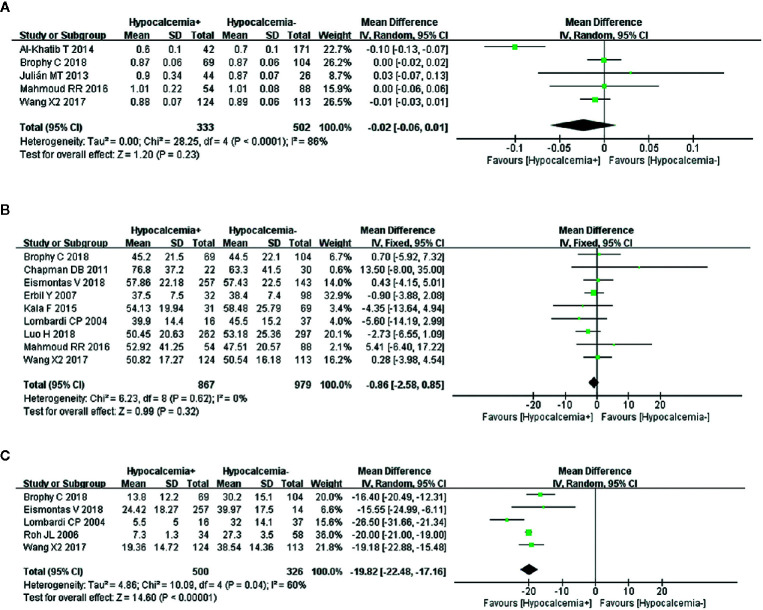
Meta-analysis results for the incidence of transient hypocalcemia between the two groups; **(A)** preoperative magnesium level for transient hypocalcemia; **(B)** preoperative PTH for transient hypocalcemia; and **(C)** postoperative 24 h PTH for transient hypocalcemia.

A random-effects model showed that the heterogeneity of the data from five studies on preoperative magnesium was significant (I^2^ = 86%, *P* < 0.0001). Transient hypocalcemia was not associated with preoperative magnesium (**Figure 5A**).

### Preoperative PTH

A fixed-effects model was used because of nonsignificant heterogeneity (I^2^ = 0%, *P* = 0.62). An analysis of nine articles, showed that preoperative PTH was not related to transient hypocalcemia (**Figure 5B**).

### Postoperative 24 h PTH

A random-effects model was used to analyze this risk factor (I^2^ = 60%, *P* = 0.04). The five studies that were analyzed showed a significant association between lower postoperative 24 h PTH and increased transient hypocalcemia (**Figure 5C**).

## Discussion

TT has become a common treatment for malignant tumors in the last few decades. It is also used to treat benign disease [e.g., multinodular goiter, GD and (Hashimoto’s) thyroiditis], in cases where the thyroid gland cannot be (partially) preserved. TT also cause several surgical complications, including postthyroidectomy hypocalcemia, hemorrhage and recurrent laryngeal nerve palsy, which are avoidable in most cases, but not all (9, 10). Among these, hypocalcemia is the most common but critical complication and requires urgent treatment because of the risks for tetany, bronchospasm and cardiac arrythmias (9). Patients with permanent hypocalcemia can be labile, and difficult to manage. They can also experience significant morbidity; however, many are maintained with a stable regimen of calcium and vitamin D (and occasionally, magnesium), which requires occasional laboratory monitoring. It is critically important for the thyroid surgeon to use strategies to prevent hypocalcemia and minimize its effects. At present, there is no effective method to treat postoperative hypocalcemia, except for calcium supplementation and 1–25 dihydroxycholecalciferol (11).

Risk factors for the development of transient hypocalcemia after TT have been investigated in many studies. Although possible risk factors have been identified in these studies, this subject has not been fully clarified. Therefore, the importance of identifying the risk factors for transient hypocalcemia, and especially, permanent hypocalcemia, necessitated this meta-analysis of the related research.

The important variable age was explored in our analysis. Many studies (12–14) have reported that hypocalcemia is associated with advanced age, whereas others (15–18) have found that younger age is a risk factor. In our study, 13 articles with 4,077 patients were analyzed to evaluate age as a predictor of transient hypocalcemia. We found that younger age was significantly more likely to be associated with hypocalcemia. Our previous studies have shown that central lymph node metastasis is more serious in younger patients with thyroid cancer. Younger patients are more likely to have CLND, which increases the rate of parathyroid injury. It might be the biological basis for hypocalcemia in younger patients.

Many studies (13, 17, 19–23) have found that postthyroidectomy hypocalcemia develops more frequently in women than in men, probably because of hormonal factors related to perimenopause. No difference was found in the incidence of hypocalcemia between premenopausal and postmenopausal women, but both groups of women had a higher incidence than that of the males (24, 25). The effects of hormonal variations on vitamin D, PTH and calcium absorption are emphasized. Consistent with these studies, 26 of the studies we included found that females had a higher incidence of transient hypocalcemia after TT.

Postthyroidectomy hypocalcemia caused by parathyroid injuries remains a difficult problem for thyroid surgeons. Parathyroid injuries include contusions, blood supply disorders, PA and IPE (26, 27). PA refers to the transplantation of the parathyroid gland, which cannot be preserved *in situ* or incised in the operation after confirmation of pathology by frozen section (28). PA has been associated with an increased risk of transient hypocalcemia at the time of the thyroidectomy. Paradoxically, routine PA may be associated with a reduced risk of permanent hypocalcemia. Lo and Lam (29) reported a higher incidence of hypocalcemia in patients who underwent PA during thyroidectomy compared to those who did not (21.4% vs. 8.1%, *P* < 0.01), but permanent hypocalcemia only occurred in the patients who did not undergo PA (1.8%). Several studies (12, 17, 20, 30) have confirmed that selective autotransplantation of one or more parathyroid glands are associated with hypocalcemia. Consistent with these results, we found that the rates of transient hypocalcemia were significantly higher in patients who underwent PA, but the incidence of permanent hypocalcemia did not increase, which confirmed that PA could fully or partially compensate for parathyroid gland abnormality and patients can recover original function after a period.

IPE is a recognized complication of thyroid surgery. Some parathyroid glands are found in intrathyroidal locations. IPE is a possible proxy of adverse patient, disease, and physician factors such as less experience on the surgeons’ side, local inflammation, and other adverse factors. Even the most experienced thyroid surgeons, however, have probably received a pathology report stating an incidental parathyroid gland was found in the thyroidectomy specimen submitted for analysis (31, 32). In the (total thyroidectomy) surgical setting, IPE increases the prevalence of hypocalcemia. In the present study, the incidence of hypocalcemia was significantly higher in the IPE group than that in the non-IPE group regardless of whether it was transient or permanent. Hypocalcemia was likely to occur when one or more parathyroid glands were excised inadvertently.

Thyroid nodules in patients with GD, local inflammation and fibrosis, have abundant blood flow and increased in intraoperative bleeding, which may obscure the surgical field of vision and increase the complexity and complications of surgery (33). Previous studies have reported a significant increase in the risk of parathyroid and recurrent laryngeal nerve injuries in patients with GD. We found that patients with GD had significantly higher incidence rates of transient and permanent hypocalcemia compared with those without this condition. Therefore, patients with GD should be monitored closely before surgery. The patients in our study were rendered euthyroid preoperatively with antithyroid medication (i.e., propylthiouracil or methimazole), and a saturated solution of potassium iodide was administered for 10 days prior to surgery to reduce the vascularity of the thyroid gland.

Previous studies have shown that patients with HT were more likely to develop hypocalcemia (34, 35). The possible reason is that the parathyroid glands in patients with HT are possibly more susceptible to injury, either due to the inflammation or due to the additional retraction required to mobilize the firmer than normal thyroid glands. In addition, HT also increases fibrosis and raises the number of small lymph nodes in the thyroidectomy field which impairs adequate parathyroid gland identification especially in the setting of an inexperienced surgeon. However, Our analysis showed no significant difference in HT between the hypocalcemia and non-hypocalcemia groups.

Hypocalcemia may develop in patients with thyroid cancer who undergo CLND, due to vascular damage or inadvertent parathyroidectomy from invasion of the thyroid capsule. The lower parathyroid glands have a higher risk for damage than the upper glands, which can usually be preserved. Injury to the lower parathyroid gland is often unavoidable in CLND (1). Therefore, patients with thyroid cancer who undergo CLND are more likely to suffer from hypocalcemia. Several studies (19, 36, 37) have reported that thyroid cancer increased the development of hypocalcemia in line with the results of our study. In our research, a significant high risk for transient and permanent hypocalcemia was observed after surgery in patients with malignant nodules.

Vitamin D is a fat-soluble vitamin derived from cholesterol. It is activated to form 25OHD in the liver and then converted to 1,25 Dihydroxyvitamin D in a PTH-dependent manner (38). Vitamin D increases the absorption of calcium from the intestinal tract, and supplementation may be helpful to patients with hypocalcemia, assuming no underlying malabsorption condition is present. Vitamin D also increases bone resorption and decreases renal excretion of calcium and phosphates (13, 39). Erbil et al. (14) showed that the risk of hypocalcemia was 558.5 times higher in patients with a 25OHD level < 37.5 nmol/L, while Al-Khatib et al. (40) found that patients with a 25OHD level < 25 nmol/L had a 7.3-times higher risk for developing laboratory hypercalcemia. Serum 25OHD >20 ng/ml was associated with a reduced risk of hypocalcemia by 72% compared to the serum level of patients with VDD (41). In our study, SVDD and VDD were associated with an increased rate of transient hypocalcemia, which is consistent with most studies. Therefore, preoperative supplementation of oral vitamin D should be considered a way of minimizing hypocalcemia in patients with SVDD and VDD.

Magnesium plays an important role in promoting calcium absorption. Hypomagnesemia has been associated with hypocalcemia in chronic disease states because it can lead to impaired PTH secretion and end-organ resistance to PTH, which together, contribute to the development of hypocalcemia (42). Our analysis of the relationship between preoperative magnesium concentration and postoperative hypocalcemia showed no significant difference in preoperative magnesium concentration between the hypocalcemia and non-hypocalcemia groups.

The value of PTH in predicting postthyroidectomy hypocalcemia has been investigated extensively and reported in the literature. PTH is the most important chemical indicator of calcium level, which usually reflects the functioning of the parathyroid gland (43). Our study found that low levels of preoperative PTH did not predict postoperative hypocalcemia. Other studies have focused on the relationship between postoperative PTH levels and postoperative hypocalcemia. The timing of PTH measurements in published studies has ranged from 10 min to 24 h postthyroidectomy, and there is no consensus on the threshold for PTH and the optimal timing for its measurement postthyroidectomy (44). Sywak et al. reported that a low 4-h postoperative PTH level (3–10 pg/ml) had a sensitivity of 90% and a specificity of 84% for predicting postoperative hypocalcemia (45). The PTH measurement on the first postoperative day has been reported to be a useful method for predicting postthyroidectomy hypocalcemia (46). In our study, a lower 24 h PTH level after surgery was a risk factor for increased transient hypocalcemia.

This study has some limitations. First, the included studies were conducted worldwide, which led to greater heterogeneity of the studies in our meta-analysis for differences in surgical methods and postoperative management. Second, surgeons with a low-volume record of thyroid surgeries increase the risk of complications after TT, which may have affected the outcomes of the included studies. Third, only a small number of studies were included in the analyses of some of the risk factors. Inconsistencies in this study’s results point to directions for future research. Multi-center trials with large samples should be conducted to identify risk factors for postoperative hypocalcemia. Fourth, because of the limitations of the included studies, we are unable to analyze the relationship between permanent hypocalcemia and age, CLND, preoperative SVDD, preoperative VDD, preoperative magnesium, preoperative PTH and postoperative 24 h PTH at present.

## Conclusion

This research found that younger age, female, PA, IPE, GD, cancer and CLND were significant clinical predictors of transient hypocalcemia, while the preoperative SVDD and VDD, and postoperative 24 h PTH were useful biochemical predictors. However, we did not find a significant difference in preoperative magnesium, PTH and HT between patients with and without hypocalcemia. For permanent hypocalcemia, IPE, GD and cancer were the clinical predictors, but gender and PA were not. Our results should be interpreted with caution because of the limited data included in this study and the relatively high heterogeneity among the studies included. Further research, including high-quality, multicenter, prospective studies and randomized trials are required to confirm our findings.

## Data Availability Statement 

The raw data supporting the conclusions of this article will be made available by the authors, without undue reservation.

## Author Contributions

HZ, YQ, WS, ZHW, WWD, LH, TZ conceived and designed the experiments. YQ and WS performed the experiments and analyzed the data. YQ wrote the article. All authors contributed to the article and approved the submitted version.

## Funding

This project was funded by the National Natural Science Foundation of China (grant number 81902726), the Postdoctoral Science Foundation of China (grant number 2018M641739) and the Natural Science Foundation of Liaoning Province (grant number 20180530090).

## Conflict of Interest

The authors declare that the research was conducted in the absence of any commercial or financial relationships that could be construed as a potential conflict of interest.

## Abbreviations

TT, Total thyroidectomy; VDD, Vitamin D deficiency; SVDD, Severe Vitamin D deficiency; PTH, Parathyroid hormone; PA, Parathyroid autotransplantation; IPE, Inadvertent parathyroid excision; GD, Graves’ disease; CLND, Central lymph node dissection; NOS, Newcastle–Ottawa Scale; OR, odds ratio; CI, confidence interval; HT, Hashimoto’s thyroiditis.
