# Supplementary material for: A Meta-Analysis of Risk Factors for Transient and Permanent Hypocalcemia After Total Thyroidectomy
Source: Front Oncol. 2021 Feb 24;10:614089. doi: 10.3389/fonc.2020.614089 (PMC7943836; doi:10.3389/fonc.2020.614089)
Supplement: Supplementary file 1 [file DataSheet_1.docx]

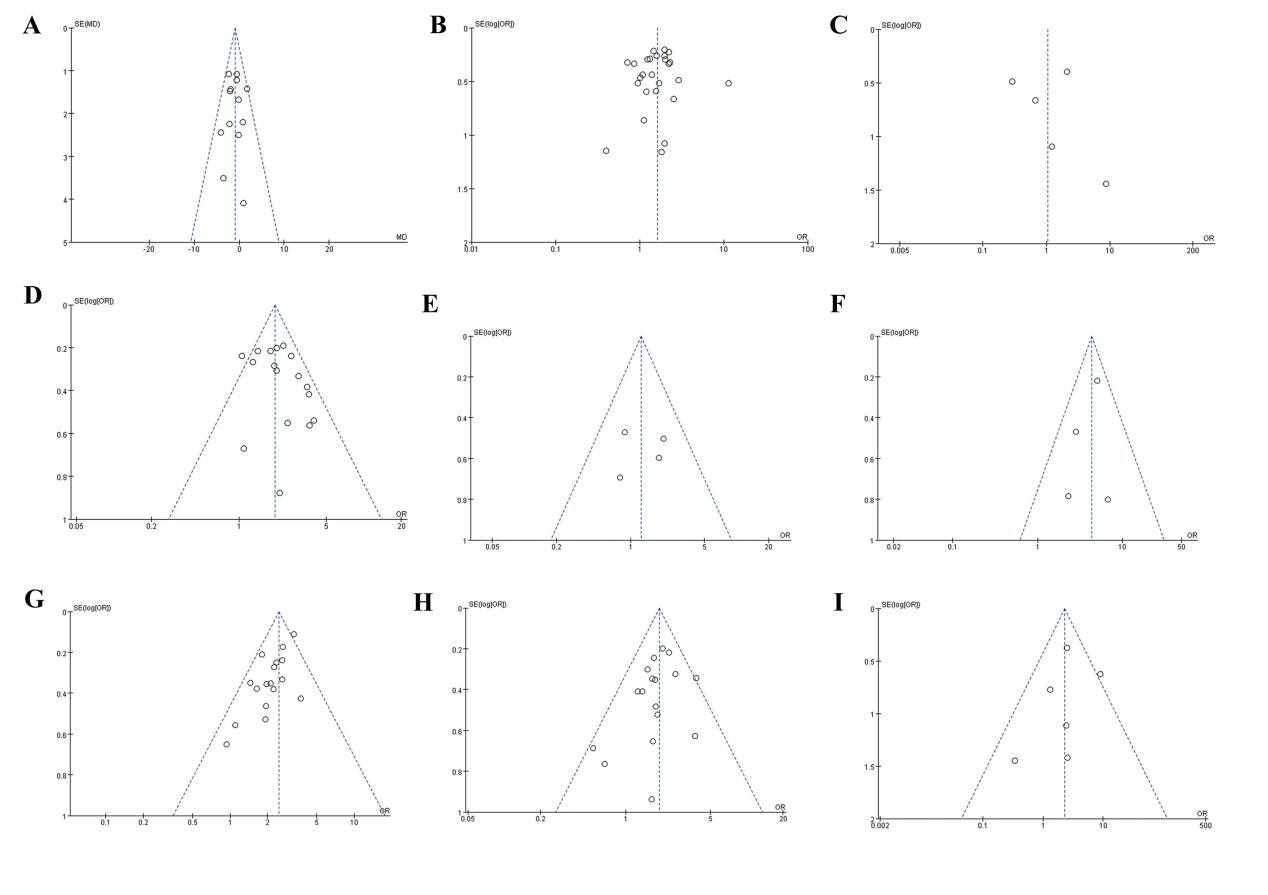


Fig. S1. Begg’s funnel plot of the association between: (A) age and hypocalcemia; (B) gender and hypocalcemia; (C) gender and permanent hypocalcemia; (D) PA and hypocalcemia; (E) PA and permanent hypocalcemia; (F) IPE and hypocalcemia; (G) IPE and permanent hypocalcemia; (H) GD and hypocalcemia; and (I) GD and permanent hypocalcemia.


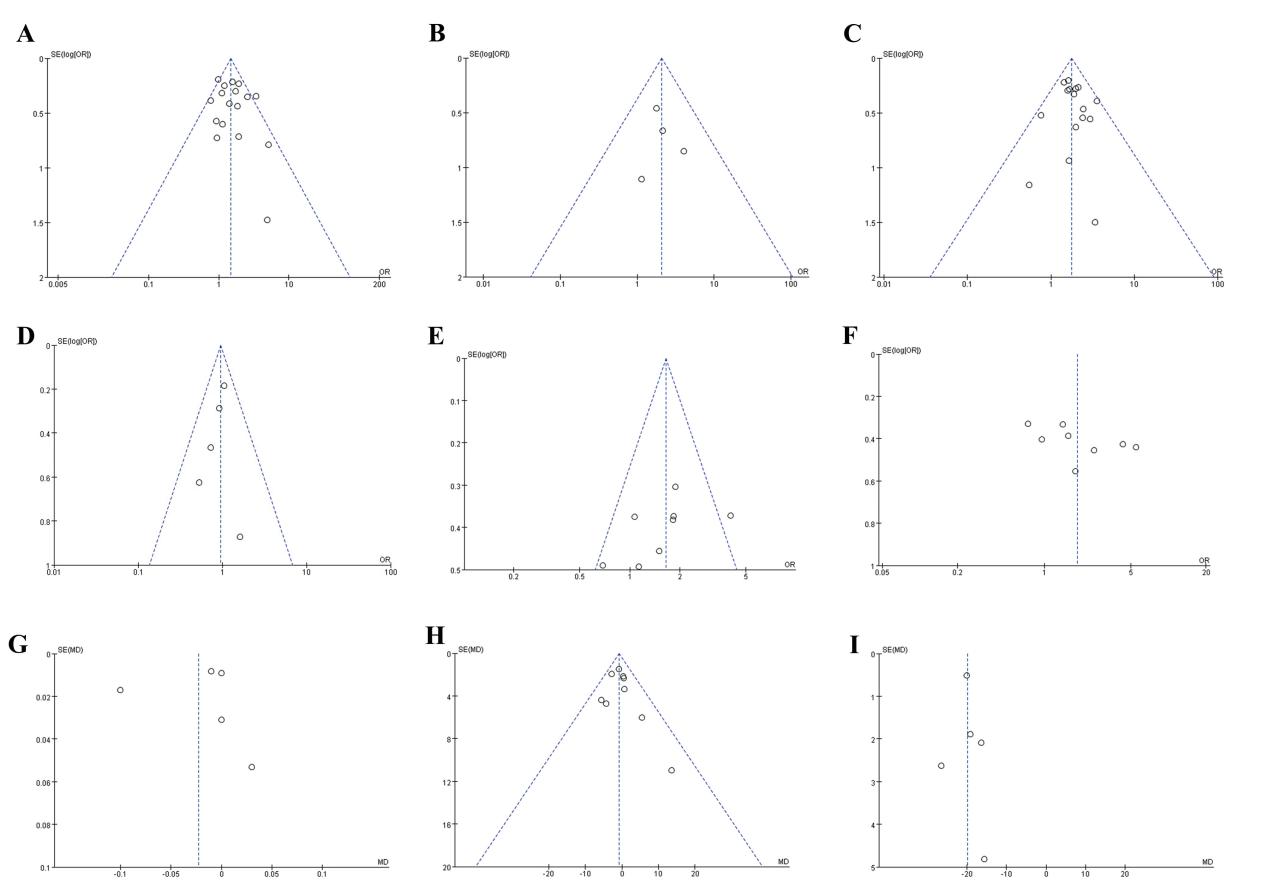


Fig. S2. Begg’s funnel plot of the association between: (A) cancer and hypocalcemia; (B) cancer and permanent hypocalcemia; (C) CLND and hypocalcemia; (D) HT and hypocalcemia; (E) SVDD and hypocalcemia; (F) VDD and hypocalcemia; (G) preoperative magnesium and hypocalcemia; (H) preoperative PTH and hypocalcemia; and (I) postoperative 24h PTH and hypocalcemia.

**Supplementary Table1. PRISMA-2009-checklist**


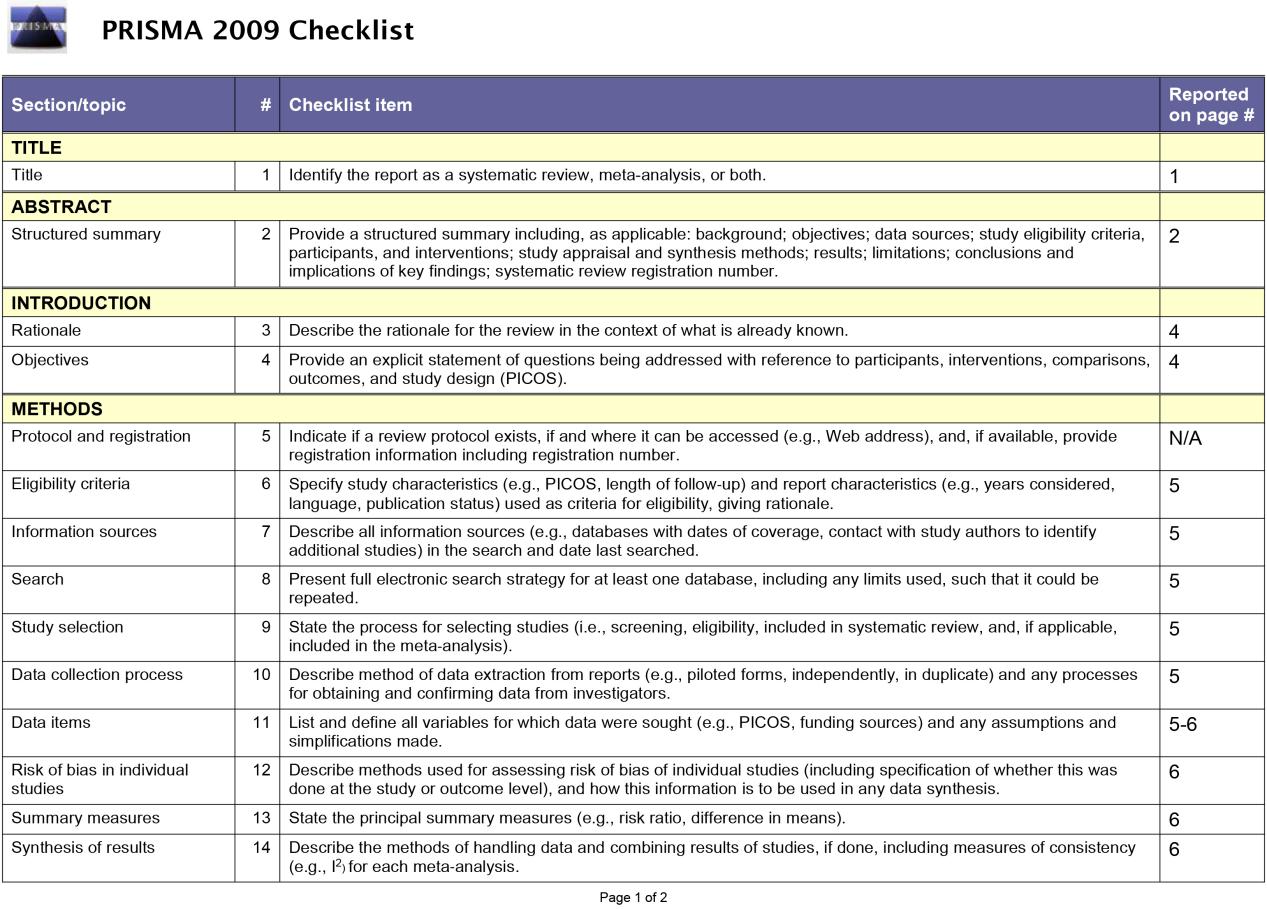

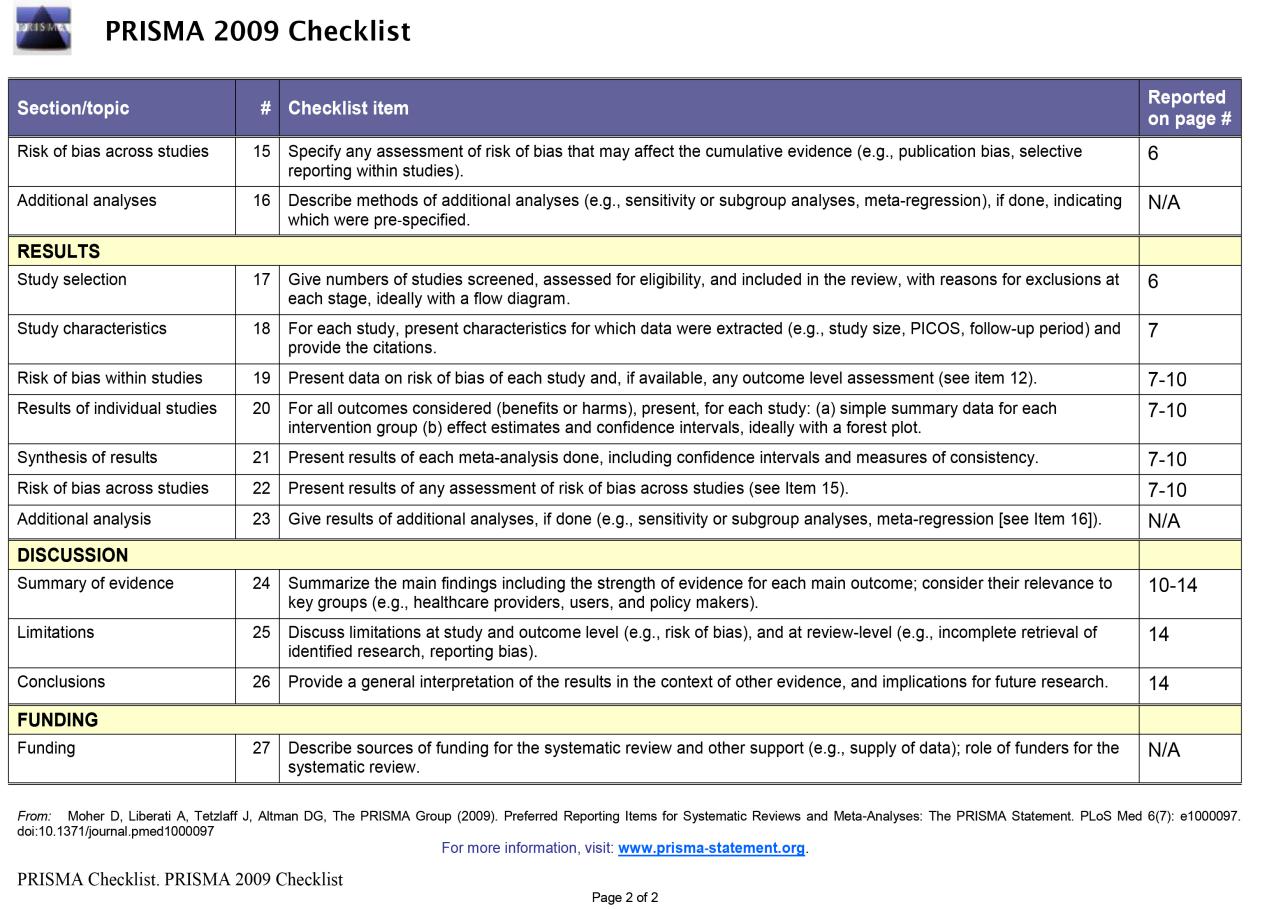


**Supplementary Table2. Included studies and the definition of hypocalcemia in the included studies.**

| **Author** | **Year** | **Country** | **Definition of hypocalcemia** |  | **Author** | **Year** | **Country** | **Definition of hypocalcemia** |
| --- | --- | --- | --- | --- | --- | --- | --- | --- |
| Arman S^[1]^ | 2018 | UK | SAC<2.1 mmol/L |  | Griffin TP^[26]^ | 2014 | Ireland | SAC<2.0 mmol/L |
| Brophy C^[2]^ | 2018 | Ireland | SC<2.0 mmol/L |  | Lorente-Poch L^[27]^ | 2014 | Spain | SC<2.0 mmol/L |
| Eismontas V^[3]^ | 2018 | Lithuania | SC<2.1 mmol/L |  | Noureldine SI^[28]^ | 2014 | USA | SC<2.1 mmol/L |
| Falch C^[4]^ | 2018 | Germany | SAC <2.0 mmol/L |  | Julián MT^[29]^ | 2013 | Spain | SC<2.1 mmol/L |
| Luo H^[5]^ | 2018 | China | SC<2.1 mmol/L |  | Lang BH^[30]^ | 2013 | China | SAC<2.1 mmol/L |
| Mazotas IG^[6]^ | 2018 | USA | SC<2.0 mmol/L |  | Nair CG^[31]^ | 2013 | India | SAC<2.0 mmol/L |
| Vasileiadis I^[7]^ | 2018 | UK | SC<2.05 mmol/L |  | Paek SH^[32]^ | 2013 | Korea | SC<2.0 mmol/L |
| Docimo G^[8]^ | 2017 | Italy | SC<2.0 mmol/L |  | Pisanu A^[33]^ | 2013 | Italy | SC<2.0 mmol/L |
| Luo H^[9]^ | 2017 | China | SC<2.1 mmol/L |  | Hallgrimsson P^[34]^ | 2012 | Sweden | SC<2.0 mmol/L |
| Sahli Z^[10]^ | 2017 | USA | SC<2.1 mmol/L |  | Lang BH^[35]^ | 2012 | China | SAC<2.1 mmol/L |
| Sitges-Serra A^[11]^ | 2017 | Spain | SC<2.0 mmol/L |  | Lin Y^[36]^ | 2012 | USA | SC<2.0 mmol/L |
| Wang X^[12]^ | 2017 | China | SAC<2.1 mmol/L |  | Chapman DB^[37]^ | 2011 | USA | SAC<2.0 mmol/L |
| Wang X2^[13]^ | 2017 | China | SAC<2.1 mmol/L |  | Kirkby-Bott J^[38]^ | 2011 | UK | SAC<2.0 mmol/l |
| Applewhite MK^[14]^ | 2016 | USA | SC<2.05 mmol/L |  | Welch KC^[39]^ | 2011 | USA | SC <2.1 mmol/L |
| Cherian AJ^[15]^ | 2016 | India | SAC<2.0 mmol/L |  | Sitges-Serra A^[40]^ | 2010 | Spain | SC<2.0 mmol/L |
| Lang BH^[16]^ | 2016 | China | SAC<2.0 mmol/L |  | Asari R^[41]^ | 2008 | Austria | SAC<2.1 mmol/L |
| Mahmoud RR^[17]^ | 2016 | Brazil | SC<2.1 mmol/L |  | Erbil Y^[42]^ | 2007 | Turkey | SC <2.0 mmol/L |
| Manatakis DK^[18]^ | 2016 | Greece | SAC<2.0 mmol/L |  | Manouras A^[43]^ | 2007 | Greece | SC <2.1 mmol/L |
| Kala F^[19]^ | 2015 | Turkey | SC<2.0 mmol/L |  | Chiang FY^[44]^ | 2006 | China | SC <2.0 mmol/L |
| Kim WW^[20]^ | 2015 | Korea | SAC<2.1 mmol/L |  | Lombardi CP^[45]^ | 2006 | Italy | SC <2.0 mmol/L |
| Lee GH^[21]^ | 2015 | Korea | SC<2.0 mmol/L |  | Roh JL^[46]^ | 2006 | Korea | SC <2.1 mmol/L |
| Miah MS^[22]^ | 2015 | UK | SC<2.1 mmol/L |  | Serpell JW^[47]^ | 2006 | Australia | SC <2.1 mmol/L |
| Puzziello A^[23]^ | 2015 | Italy | SC<2.0 mmol/L |  | Palazzo FF^[48]^ | 2005 | Australia | SAC<2.0 mmol/L |
| Al-Khatib T^[24]^ | 2014 | Saudi Arabia | SAC<2.0 mmol/L |  | Lombardi CP^[49]^ | 2004 | Italy | SC <2.0 mmol/L |
| Edafe O^[25]^ | 2014 | UK | SAC<2.1 mmol/L |  | Thomusch O^[50]^ | 2003 | Germany | SC <2.0 mmol/L |

**Abbreviation**: SAC, serum adjusted calcium; SC, serum calcium

**Included studies**

[1] Arman S, Vijendren A, Mochloulis G. The incidence of post-thyroidectomy hypocalcaemia: a retrospective single-centre audit. *Ann R Coll Surg Engl*. 2019;101(4):273-278.

[2] Brophy C, Woods R, Murphy MS, Sheahan P. Perioperative magnesium levels in total thyroidectomy and relationship to hypocalcemia. *Head Neck*. 2019.

[3] Eismontas V, Slepavicius A, Janusonis V, Zeromskas P, Beisa V, Strupas K, Dambrauskas Z, Gulbinas A, Martinkenas A. Predictors of postoperative hypocalcemia occurring after a total thyroidectomy: results of prospective multicenter study. BMC Surg. 2018. 18(1): 55-.

[4] Falch C, Hornig J, Senne M, Braun M, Konigsrainer A, Kirschniak A, Muller S. Factors predicting hypocalcemia after total thyroidectomy - A retrospective cohort analysis. Int J Surg. 2018. 55: 46-50.

[5] Luo H, Zhao W, Yang H, Su A, Wang B, Zhu J, AUID- O. In Situ Preservation Fraction of Parathyroid Gland in Thyroidectomy: A Cohort Retrospective Study. Int J Endocrinol. 2018. 2018: 7493143-.

[6] Mazotas IG, Yen T, Park J, Liu Y, Eastwood DC, Carr AA, Evans DB, Wang TS. A postoperative parathyroid hormone-based algorithm to reduce symptomatic hypocalcemia following completion/total thyroidectomy: A retrospective analysis of 591 patients. Surgery. 2018. 164(4): 746-753.

[7] Vasileiadis I, Charitoudis G, Vasileiadis D, Kykalos S, Karatzas T. Clinicopathological characteristics of incidental parathyroidectomy after total thyroidectomy: The effect on hypocalcemia. A retrospective cohort study. Int J Surg. 2018. 55: 167-174.

[8] Docimo G, Ruggiero R, Casalino G, Del GG, Docimo L, Tolone S, AUID- OHOO. Risk factors for postoperative hypocalcemia. Updates Surg. 2017. 69(2): 255-260-.

[9] Luo H, Yang H, Zhao W, Wei T, Su A, Wang B, Zhu J. Hypomagnesemia predicts postoperative biochemical hypocalcemia after thyroidectomy. BMC Surg. 2017. 17(1): 62.

[10] Sahli Z, Najafian A, Kahan S, Schneider EB, Zeiger MA, Mathur A. One-Hour Postoperative Parathyroid Hormone Levels Do Not Reliably Predict Hypocalcemia After Thyroidectomy. *World J Surg*. 2018;42(7):2128-2133.

[11] Sitges-Serra A, Gallego-Otaegui L, Suárez S, Lorente-Poch L, Munné A, Sancho JJ. Inadvertent parathyroidectomy during total thyroidectomy and central neck dissection for papillary thyroid carcinoma. Surgery. 2017. 161(3): 712-719.

[12] Wang X, Zhu J, Liu F, Gong Y, Li Z. Preoperative vitamin D deficiency and postoperative hypocalcemia in thyroid cancer patients undergoing total thyroidectomy plus central compartment neck dissection. Oncotarget. 2017. 8(44): 78113-78119.

[13] Wang X, Zhu J, Liu F, Gong Y, Li Z. Postoperative hypomagnesaemia is not associated with hypocalcemia in thyroid cancer patients undergoing total thyroidectomy plus central compartment neck dissection. International journal of surgery (London, England). 2017. 39: 192-196-.

[14] Applewhite MK, White MG, Xiong M, Pasternak JD, Abdulrasool L, Ogawa L, Suh I, Gosnell JE, Kaplan EL, Duh QY, Angelos P, Shen WT, Grogan RH. Incidence, Risk Factors, and Clinical Outcomes of Incidental Parathyroidectomy During Thyroid Surgery. Ann Surg Oncol. 2016. 23(13): 4310-4315.

[15] Cherian AJ, Ponraj S, Gowri S M, Ramakant P, Paul TV, Abraham DT, Paul MJ. The role of vitamin D in post-thyroidectomy hypocalcemia: Still an enigma. Surgery. 2016. 159(2): 532-8.

[16] Lang BH, Chan DT, Chow FC. Visualizing fewer parathyroid glands may be associated with lower hypoparathyroidism following total thyroidectomy. Langenbecks Arch Surg. 2016. 401(2): 231-8.

[17] Mahmoud RR, Neto VJ, Alves W, Lin CS, Leite AK, Matos LL, Filho VJ, Cernea CR. Hypomagnesemia associated with hypocalcemia after total thyroidectomy: an observational study. Magnes Res. 2016. 29(2): 43-7.

[18] Manatakis DK, AUID- O, Balalis D, Soulou VN, Korkolis DP, Plataniotis G, Gontikakis E. Incidental Parathyroidectomy during Total Thyroidectomy: Risk Factors and Consequences. Int J Endocrinol. 2016. 2016: 7825305-.

[19] Kala F, Sarici IS, Ulutas KT, Sevim Y, Dogu A, Sarigoz T, Tastan B, Topuz O, Ertan T. Intact parathormone measurement 1 hour after total thyroidectomy as a predictor of symptomatic hypocalcemia. Int J Clin Exp Med. 2015. 8(10): 18813-8-.

[20] Kim WW, Chung SH, Ban EJ, Lee CR, Kang SW, Jeong JJ, Nam KH, Chung WY, Park CS. Is Preoperative Vitamin D Deficiency a Risk Factor for Postoperative Symptomatic Hypocalcemia in Thyroid Cancer Patients Undergoing Total Thyroidectomy Plus Central Compartment Neck Dissection. Thyroid. 2015. 25(8): 911-8.

[21] Lee GH, Ku YH, Kim HI, Lee MC, Kim MJ. Vitamin D level is not a predictor of hypocalcemia after total thyroidectomy. Langenbecks Arch Surg. 2015. 400(5): 617-22.

[22] Miah MS, Mahendran S, Mak C, Leese G, Smith D. Pre-operative serum alkaline phosphatase as a predictive indicator of post-operative hypocalcaemia in patients undergoing total thyroidectomy. J Laryngol Otol. 2015. 129(11): 1128-32.

[23] Puzziello A, Gervasi R, Orlando G, Innaro N, Vitale M, Sacco R. Hypocalcaemia after total thyroidectomy: could intact parathyroid hormone be a predictive factor for transient postoperative hypocalcemia. Surgery. 2015. 157(2): 344-8.

[24] Al-Khatib T, Althubaiti AM, Althubaiti A, Mosli HH, Alwasiah RO, Badawood LM. Severe vitamin D deficiency: a significant predictor of early hypocalcemia after total thyroidectomy. Otolaryngol Head Neck Surg. 2015. 152(3): 424-31.

[25] Edafe O, Prasad P, Harrison BJ, Balasubramanian SP. Incidence and predictors of post-thyroidectomy hypocalcaemia in a tertiary endocrine surgical unit. Ann R Coll Surg Engl. 2014. 96(3): 219-23.

[26] Griffin TP, Murphy MS, Sheahan P. Vitamin D and risk of postoperative hypocalcemia after total thyroidectomy. JAMA Otolaryngol Head Neck Surg. 2014. 140(4): 346-51.

[27] Lorente-Poch L, Sancho JJ, Ruiz S, Sitges-Serra A. Importance of in situ preservation of parathyroid glands during total thyroidectomy. Br J Surg. 2015. 102(4): 359-67.

[28] Noureldine SI, Genther DJ, Lopez M, Agrawal N, Tufano RP. Early predictors of hypocalcemia after total thyroidectomy: an analysis of 304 patients using a short-stay monitoring protocol. *JAMA Otolaryngol Head Neck Surg*. 2014;140(11):1006-1013.

[29] Julián MT, Balibrea JM, Granada ML, Moreno P, Alastrué A, Puig-Domingo M, Lucas A. Intact parathyroid hormone measurement at 24 hours after thyroid surgery as predictor of parathyroid function at long term. Am J Surg. 2013. 206(5): 783-9.

[30] Lang BH, Wong KP, Cheung CY, Fong YK, Chan DK, Hung GK. Does preoperative 25-hydroxyvitamin D status significantly affect the calcium kinetics after total thyroidectomy. *World J Surg*. 2013;37(7):1592-1598.

[31] Nair CG, Babu MJ, Menon R, Jacob P. Hypocalcaemia following total thyroidectomy: An analysis of 806 patients. Indian J Endocrinol Metab. 2013. 17(2): 298-303-.

[32] Paek SH, Lee YM, Min SY, Kim SW, Chung KW, Youn YK. Risk factors of hypoparathyroidism following total thyroidectomy for thyroid cancer. World J Surg. 2013. 37(1): 94-101.

[33] Pisanu A, Saba A, Coghe F, Uccheddu A. Early prediction of hypocalcemia following total thyroidectomy using combined intact parathyroid hormone and serum calcium measurement. *Langenbecks Arch Surg*. 2013;398(3):423-430.

[34] Hallgrimsson P, Nordenström E, Bergenfelz A, Almquist M. Hypocalcaemia after total thyroidectomy for Graves' disease and for benign atoxic multinodular goitre. Langenbecks Arch Surg. 2012. 397(7): 1133-7.

[35] Lang BH, Yih PC, Ng KK. A prospective evaluation of quick intraoperative parathyroid hormone assay at the time of skin closure in predicting clinically relevant hypocalcemia after thyroidectomy. World J Surg. 2012. 36(6): 1300-6.

[36] Lin Y, Ross HL, Raeburn CD, DeWitt PE, Albuja-Cruz M, Jones EL, McIntyre RC Jr. Vitamin D deficiency does not increase the rate of postoperative hypocalcemia after thyroidectomy. *Am J Surg*. 2012;204(6):888-893; discussion 893-894.

[37] Chapman DB, French CC, Leng X, Browne JD, Waltonen JD, Sullivan CA. Parathyroid hormone early percent change: an individualized approach to predict postthyroidectomy hypocalcemia. Am J Otolaryngol. 2012. 33(2): 216-20.

[38] Kirkby-Bott J, Markogiannakis H, Skandarajah A, Cowan M, Fleming B, Palazzo F. Preoperative vitamin D deficiency predicts postoperative hypocalcemia after total thyroidectomy. *World J Surg*. 2011;35(2):324-330.

[39] Welch KC, McHenry CR. Total thyroidectomy: is morbidity higher for Graves' disease than nontoxic goiter. J Surg Res. 2011. 170(1): 96-9.

[40] Sitges-Serra A, Ruiz S, Girvent M, Manjón H, Dueñas JP, Sancho JJ. Outcome of protracted hypoparathyroidism after total thyroidectomy. Br J Surg. 2010. 97(11): 1687-95.

[41] Asari R, Passler C, Kaczirek K, Scheuba C, Niederle B. Hypoparathyroidism after total thyroidectomy: a prospective study. Arch Surg. 2008. 143(2): 132-7; discussion 138.

[42] Erbil Y, Bozbora A, Ozbey N, Issever H, Aral F, Ozarmagan S, Tezelman S. Predictive value of age and serum parathormone and vitamin d3 levels for postoperative hypocalcemia after total thyroidectomy for nontoxic multinodular goiter. Arch Surg. 2007. 142(12): 1182-7.

[43] Manouras A, Markogiannakis H, Lagoudianakis E, Antonakis P, Genetzakis M, Papadima A, Konstantoulaki E, Papanikolaou D, Kekis P. Unintentional parathyroidectomy during total thyroidectomy. Head Neck. 2008. 30(4): 497-502.

[44] Chiang FY, Lin JC, Wu CW, Lee KW, Lu SP, Kuo WR, Wang LF. Morbidity after total thyroidectomy for benign thyroid disease: comparison of Graves' disease and non-Graves' disease. Kaohsiung J Med Sci. 2006. 22(11): 554-9.

[45] Lombardi CP, Raffaelli M, Princi P, Dobrinja C, Carrozza C, Di Stasio E, D'Amore A, Zuppi C, Bellantone R. Parathyroid hormone levels 4 hours after surgery do not accurately predict post-thyroidectomy hypocalcemia. Surgery. 2006. 140(6): 1016-23; discussion 1023-5.

[46] Roh JL, Park CI. Intraoperative parathyroid hormone assay for management of patients undergoing total thyroidectomy. Head Neck. 2006. 28(11): 990-7.

[47] Serpell JW, Phan D. Safety of total thyroidectomy. ANZ J Surg. 2007. 77(1-2): 15-9.

[48] Palazzo FF, Sywak MS, Sidhu SB, Barraclough BH, Delbridge LW. Parathyroid autotransplantation during total thyroidectomy--does the number of glands transplanted affect outcome. *World J Surg*. 2005;29(5):629-631.

[49] Lombardi CP, Raffaelli M, Princi P, Santini S, Boscherini M, De Crea C, Traini E, D'Amore AM, Carrozza C, Zuppi C, Bellantone R. Early prediction of postthyroidectomy hypocalcemia by one single iPTH measurement. Surgery. 2004. 136(6): 1236-41.

[50] Thomusch O, Machens A, Sekulla C, Ukkat J, Brauckhoff M, Dralle H. The impact of surgical technique on postoperative hypoparathyroidism in bilateral thyroid surgery: a multivariate analysis of 5846 consecutive patients. Surgery. 2003. 133(2): 180-5.
